# Ultrasound-guided femoral vascular access in adult congenital heart disease patients undergoing catheter ablation on uninterrupted oral anticoagulation

**DOI:** 10.1007/s00392-026-02935-1

**Published:** 2026-06-02

**Authors:** Marta Telishevska, Sarah Lengauer, Theresa Reiter, Hannah Krafft, Miruna Popa, Fabian Bahlke, Florian Englert, Nico Erhard, Isabel Deisenhofer, Gabriele Hessling

**Affiliations:** German Heart Center Munich, Munich, Germany

**Keywords:** Ultrasound-guided vascular access, Adult congenital heart disease, Catheter ablation, Atrial arrhythmias, Vascular access complications

## Abstract

**Background:**

Data on ultrasound-guided vascular access (UGVA) to reduce vascular access complications in adult congenital heart disease (ACHD) patients undergoing catheter ablation (CA) on uninterrupted oral anticoagulation (OAC) are limited.

**Objective:**

Comparison of UGVA to a standard approach with regard to vascular access complications in ACHD patients.

**Methods:**

We retrospectively analyzed 477 consecutive ACHD patients (52% male, mean age 46 ± 14 years) with simple (*n* = 166), moderate (*n* = 133), and complex (*n* = 178) congenital heart disease (CHD) who underwent 639 CA procedures for atrial tachycardia or fibrillation between 2014 and 2024. UGVA (243 patients, 271 ablations) was compared to a conventional approach (234 patients, 368 ablations) regarding vascular access complications under uninterrupted OAC (DOAC or VKA).

**Results:**

Access was obtained via the femoral vein (*n* = 321), both femoral vein and artery (*n* = 311), or jugular vein with femoral artery (*n* = 7). UGVA significantly reduced overall vascular access complications compared to the standard approach (4.1% vs. 13.5%; *p* < 0.001). Major complications occurred only in the conventional group (*n* = 4; AV fistula = 2, retroperitoneal hematoma = 2; *p* = 0.086). Minor complications—hematomas > 5 cm, small AV fistulas treated conservatively, or pseudoaneurysms treated by compression or ultrasound-guided thrombin injection—were also significantly lower with UGVA (4.1% vs. 12.5%; *p* < 0.001).

**Conclusions:**

UGVA significantly decreases vascular access complications in ACHD patients undergoing CA on uninterrupted OAC and may be advantageous for routine use in clinical practice.

**Graphical Abstract:**

**Figure:**
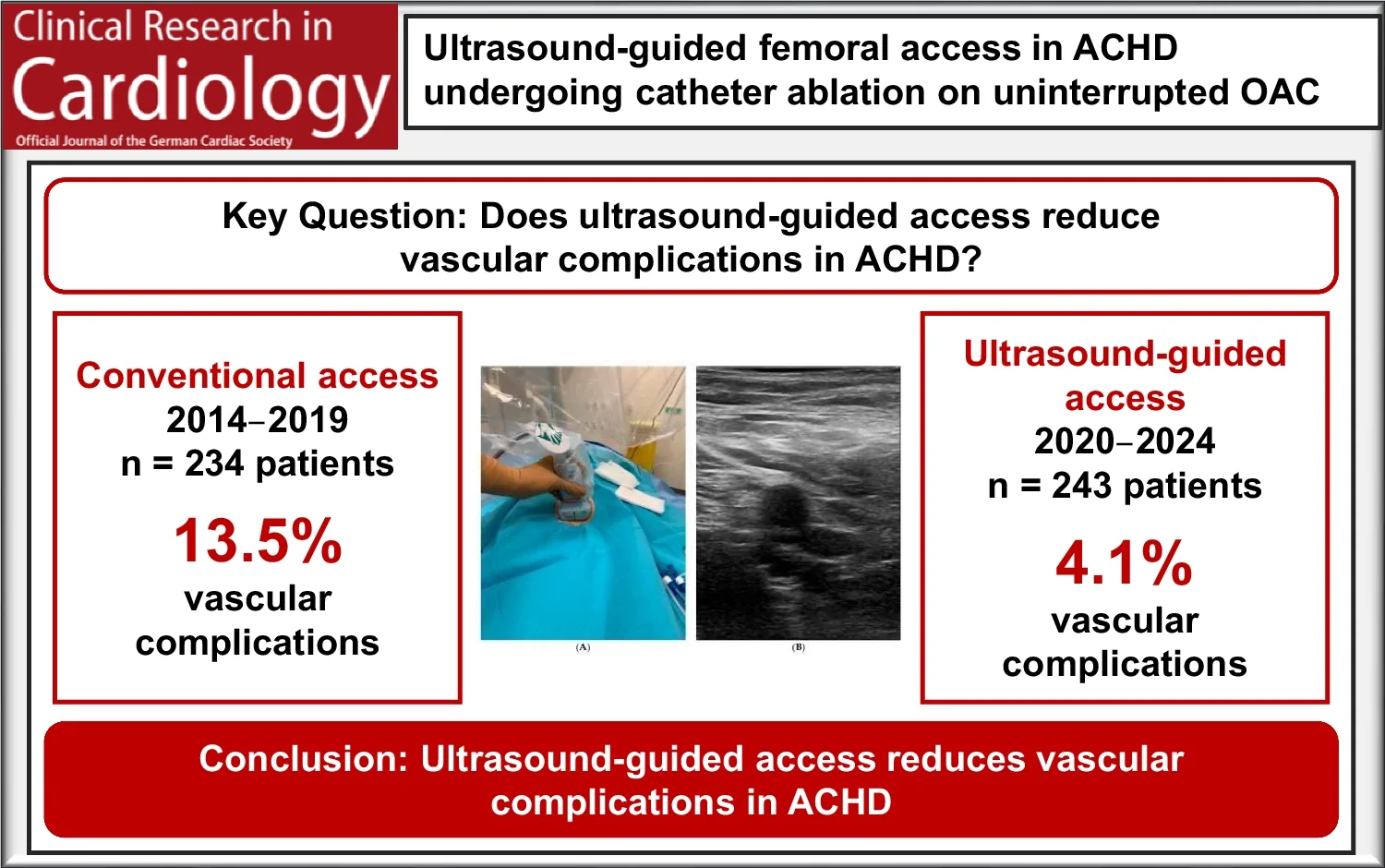

**Supplementary Information:**

The online version contains supplementary material available at https://doi.org/10.1007/s00392-026-02935-1.

## Introduction

Cardiac arrhythmias in ACHD patients have increasingly come into focus over the last two decades due to an ageing patient population with an earlier arrhythmia onset compared to patients without CHD [1–4]. CA for atrial arrhythmias is recommended as first-line therapy in ACHD patients regardless of CHD complexity [5, 6]. However, cardiac anatomy and previous cardiac surgery might present technical challenges and a higher recurrence and redo ablation rate has been described [7].

Vascular complications are among the most common complications of CA as procedures are performed under oral anticoagulation and heparin and the insertion of multiple sheaths is required. ACHD patients have often undergone (multiple) previous cardiac catheters or electrophysiological studies with vascular access using the groin vessels. The incidence of major vascular complications in non ACHD is reported in the range of 0.2% to 1.5% [8–10]. In patients with atrial fibrillation (AF) without CHD, vascular complications rates are reported between 2 and 4% [11]. Recent data in non-ACHD patients suggest in non-randomized trials that ultrasound-guided vascular access (UGVA) is a useful method to reduce major and minor vascular complications [12–14]. It allows direct visualization of venous and arterial anatomy and reveals anatomical variants. In an earlier study from our group with ACHD patients on uninterrupted oral anticoagulation undergoing CA for atrial arrhythmias, we had observed a major bleeding/vascular access complication rate of 1.1% and a minor complication rate of 12.5% [15]. Based on this experience, we changed our conventional femoral access approach to a UGVA strategy in 2020. Here, we report our outcomes before and after this change of practice.

## Methods

### Study population and design

A retrospective analysis of 477 consecutive ACHD patients (52% male, mean age 46 14 years) who underwent 639 catheter ablation procedures for atrial flutter/tachycardia or AF between 2014 and 2024 was performed. In 2020, we had changed our clinical practice from a conventional vascular access approach to UGVA. UGVA was used in 243 patients (51%) with 271 ablations (42%) and compared to the conventional approach in 234 patients (49%) with 368 ablations (58%) with regard to vascular access complications. Clinical data, including arrhythmia type, medical history, OAC regimen, ablation strategy, and peri-procedural complications, were extracted from the institutional database. Medical history included heart failure (LV ejection fraction ≤ 45%), CHA_2DS_2-VASc score, hypertension, diabetes, prior stroke/TIA, vascular/coronary disease, and body mass Index. All ablations were performed on uninterrupted OAC (DOAC or VKA). Arrhythmias were defined as paroxysmal or persistent AF and typical (cavo-tricuspid isthmus dependent) or atypical atrial flutter/atrial tachycardia. The UGVA group (patients treated from 2020 onward) underwent fewer ablation procedures per patient (on average ~ 1.12 procedures/patient) compared to the conventional group (2014–2019 era, ~ 1.57 procedures/patient). This discrepancy arises largely from the longer follow-up period in the conventional group: patients in the earlier cohort had more time to experience arrhythmia recurrences and thus return for repeat ablations. Additionally, some complex ACHD patients in the conventional cohort underwent multiple procedures before 2020.

The study was approved by the institutional ethics review board.

### Vascular access strategies

All patients were treated according to an identical institutional protocol, ensuring procedural comparability. For femoral venous access, all patients received either one 6 F and two 8 F sheaths or three 8 F sheaths. In cases requiring transseptal or baffle puncture or right atrial ablation in markedly enlarged atria, a 12.8F Agilis™ sheath was used. Arterial access consisted of a routine 4 F sheath for invasive blood pressure monitoring or, if retrograde left-sided access was necessary, an 8 F arterial sheath. Venous access priority was the right femoral vein (2–3 sheaths). In case of right femoral vein occlusion, the left femoral vein was used. In patients with bilateral femoral venous occlusion (*n* = 7), an alternative venous route (e.g., internal jugular) combined with femoral arterial access was used.

*Conventional approach:* Anatomical landmark-guided puncture using the Seldinger technique. The puncture site was chosen based on surface landmarks of the inguinal ligament and femoral arterial pulsation. Fluoroscopy was not used to guide the skin puncture but was permitted to confirm guidewire/sheath intraluminal position when necessary.

*Ultrasound-guided approach:*Color Doppler and two-dimensional real-time ultrasound (SonoSite S-II, Fujifilm, Bothell, WA, USA) were used to guide femoral venous and arterial access. An 8‑MHz linear array transducer in sterile sheath was placed perpendicular to the vessel course. The femoral vein was differentiated from the artery by compressibility and color Doppler. Vascular cannulation was performed under direct visualization of the needle entering the vessel using the Seldinger technique. The bifurcation of the common femoral artery into the superficial and profunda femoris branches was identified, and access was obtained above the bifurcation, where the femoral vein is typically medial to the artery [16].

### Procedure and peri-procedural management

All patients underwent duplex ultrasound of the groin vessels on the day before ablation to exclude major anomalies. Cardiac CT angiography was performed to evaluate intracardiac anatomy and procedural planning; groin vessels were not routinely visualized on cardiac CT. Ablations were performed under deep IV sedation (fentanyl/propofol). In moderate/complex CHD, invasive arterial pressure monitoring (4F sheath) was used. For AF or left-sided AT, a single TSP with double transseptal access was performed followed by weight-adjusted heparin bolus and infusion (50–60 U/kg/h) titrated to ACT: 200–250 s for right atrial ablation; > 300 s for left atrial ablation (ACT checked every 30 min). If TSP or trans-baffle puncture (patients after Mustard/Senning procedure) was not performed, retrograde arterial access (8F) was used with a magnetic navigation system (Stereotaxis Inc, St. Louis, MO) when applicable. All patients with paroxysmal AF underwent antral circumferential pulmonary vein isolation using radiofrequency energy and standard 7 F (4—mm) irrigated-tip ablation catheters. In patients with persistent AF, an additional ablation of complex fractionated atrial electrograms was performed. In patients with typical atrial flutter, ablation of the cavotricuspid isthmus was performed using a 7 F non-irrigated (8—mm tip) or 7 F irrigated-tip (4—mm) ablation catheter. In patients with atypical atrial flutter/atrial tachycardia, linear lesions or focal ablation was performed according to the underlying tachycardia mechanism. Procedures were performed using a 3-dimensional mapping system (*n* = 331 CARTO®, Biosense Webster, Diamond Bar, CA; *n* = 210 EnSite NavX®, Abbott; *n* = 22 Rhythmia HDx®, Boston Scientific Q5; *n* = 76 Stereotaxis Niobe®/CARTO®). In 6 cases, no 3D mapping system was used. If available, computer tomography scans were integrated into the mapping system to display anatomy. No protamine was administered at the end of the ablation. In all cases, a purse-string suture was applied to venous puncture sites. Venous sheaths were removed directly after purse-string suture, and a groin compression bandage was applied for 6 h in patients; for arterial punctures, a vascular closure system (FemoSeal®, Angio-Seal®) was used. Closure and sheath strategies were uniform in both groups. Post-procedural ultrasound policy: routine duplex ultrasound of the puncture site was performed before discharge in all patients. All patients underwent routine pre- and post-procedural duplex ultrasound of the femoral vessels to identify even minor vascular abnormalities. Post-procedural inpatient monitoring was performed for one to two nights. All patients had daily clinical examinations during hospitalization and were instructed to return for any vascular symptoms after discharge.

### Study endpoints

Primary endpoints were the occurrence of vascular complications peri- or postinterventionally. Major complications were defined as AV fistulas, pseudoaneurysms, or retroperitoneal hematoma requiring treatment such as transfusion, intervention, surgical treatment, prolonged mechanical compression, analgesics, or extended hospitalization. Minor complications were defined as hematomas > 5 cm without intervention, AV fistulas treated conservatively or pseudoaneurysms treated by manual compression or ultrasound-guided thrombin injection.

### Statistical analysis

Continuous variables are presented as mean ± SD and compared by two-sided *t*‑test or Mann–Whitney *U* test, as appropriate. Categorical variables were compared by Fisher’s exact test or chi-square test (Yates correction where indicated). A *p*‑value < 0.05 was considered significant. To address baseline imbalances, pre-specified sensitivity analyses were added. A multivariate logistic regression analysis was performed with UGVA as the primary independent variable, adjusting for age, sex, BMI, DOAC versus VKA use, arterial hypertension, and CHD complexity. Odds ratios with 95% confidence intervals were calculated. For the propensity score analysis, a score was generated for each patient based on baseline variables, particularly anticoagulant type and hypertension. Sensitivity analyses using propensity score adjustment were then conducted to account for significant group differences and reduce potential confounding. All analyses were performed using SPSS 27.0 (IBM, Chicago, IL, USA).

## Results

### Baseline characteristics

Baseline characteristics and CHD complexity are presented in Table 1, arrhythmia types in Table 2. Groups were broadly comparable; however, DOAC/VKA distribution differed significantly (chi-square with Yates correction *p* = 0.021; without correction *p* = 0.017), and arterial hypertension differed without Yates’ correction (*p* = 0.047; with correction *p* = 0.060). In a multivariate logistic regression model including UGVA as the main variable and adjusting for DOAC versus VKA use, age, BMI, CHD complexity, and number of prior procedures, UGVA remained independently associated with a lower risk of vascular access complications (OR 0.32, 95% CI 0.14–0.68, *p* = 0.003). The propensity score-matched dataset (*n* = 230 pairs) demonstrates that all measured baseline characteristics except arterial hypertension were well balanced. The arterial hypertension was more common in the UGVA group. In the propensity score–matched dataset of 120 pairs, the UGVA and conventional groups were well balanced with respect to anticoagulant type (DOAC 69 vs. 66; VKA 51 vs. 54; *p* = 0.79). Constructing 230 matched pairs with the real data would only be possible by accepting poor matches, which contradicts the fundamental goal of propensity score matching to achieve good covariate balance and minimize confounding. Therefore, we restricted the analysis to 120 well-matched pairs, as the available data support adequate overlap and balance only up to this number; attempting to force more pairs would compromise the validity of the analysis. The results and baseline characteristics of the propensity score-matched cohort have been added to the Supplementary Table 1, allowing detailed comparison of clinical endpoints in this subgroup.

**Table 1:** Patient characteristics in 477 ACHD patients

Variable	Total *n* = 477	UGVA *n* = 243	Conventional *n* = 234	*p*-value
Age (years)	46 ± 14	44 ± 14	48 ± 14	n.s
Gender (male)	248 (52)	119 (49)	131 (56)	n.s
BMI (kg/m^2^)	26 ± 5	27 ± 5	26 ± 5	n.s
Arterial hypertension	124 (26)	70 (29)	49 (21)	0.047
Diabetes	30 (6.4)	14 (6)	16 (7)	n.s
History of stroke/TIA	32 (6.8)	12 (5)	21 (9)	n.s
CAD	30 (6.4)	14 (6)	16 (7)	n.s
DOAC use	281 (59)	156 (64)	125 (53)	0.017
VKA use	196 (41)	87 (36)	109 (47)	0.017
Simple CHD	*n* = 166 (35)	77 (46)	89 (54)	n.s
Moderate CHD	*n* = 133 (28)	70 (53)	63 (47)	n.s
Complex CHD	*n* = 178 (37)	96 (54)	82 (46)	n.s

Continuous values are expressed as mean ± standard deviation. Categorial values are expressed as number and percentage. Values are given as *n* (%) unless otherwise indicated

*TIA* transient ischemic attack, *CAD* coronary artery disease, *BMI* body mass index; *DOAC* direct oral anticoagulant. *Statistically significant results, *p* < 0.05, *n.s.* non-significant

**Table 2:** Type of arrhythmia in 639 ablation cases

Cardiac arrhythmia	Total *n* = 639	UGVA *n* = 271	Conventional *n* = 368	*p*-value
Atrial flutter	281 (44)	108 (40)	173 (47)	n.s
Atrial fibrillation	237 (37)	103 (38)	134 (36)	n.s
Atrial fibrillation/flutter	121 (19)	60 (22)	61 (16)	n.s

Values are given as n (%) unless otherwise indicated

### Oral anticoagulation

A DOAC was administered in 281 patients (59%) including apixaban (*n* = 160 [57%]), rivaroxaban (*n* = 84 [30%]), edoxaban (*n* = 28 [10%]), or dabigatran (*n* = 9 [3%]). VKA was administered in 196 patients (41%), including phenprocoumon (*n* = 162 [83%]) or warfarin (*n* = 34 [17%]) with a INR value of 2–3. A significant difference was found regarding the use of DOACs and VKAs between groups (DOAC 64% vs. 53%, *p* = 0.017; VKA 36% vs. 47%, *p* = 0.017), consistent with Table 1 or VKA in the UGVA (36% versus 47%; *p* > 0.05). In the final propensity score–matched dataset of 120 pairs, the UGVA and conventional groups were well balanced with respect to anticoagulant type (DOAC 69 vs. 66; VKA 51 vs. 54; *p* = 0.79).

### Vascular access complications

**Fig. 1:**
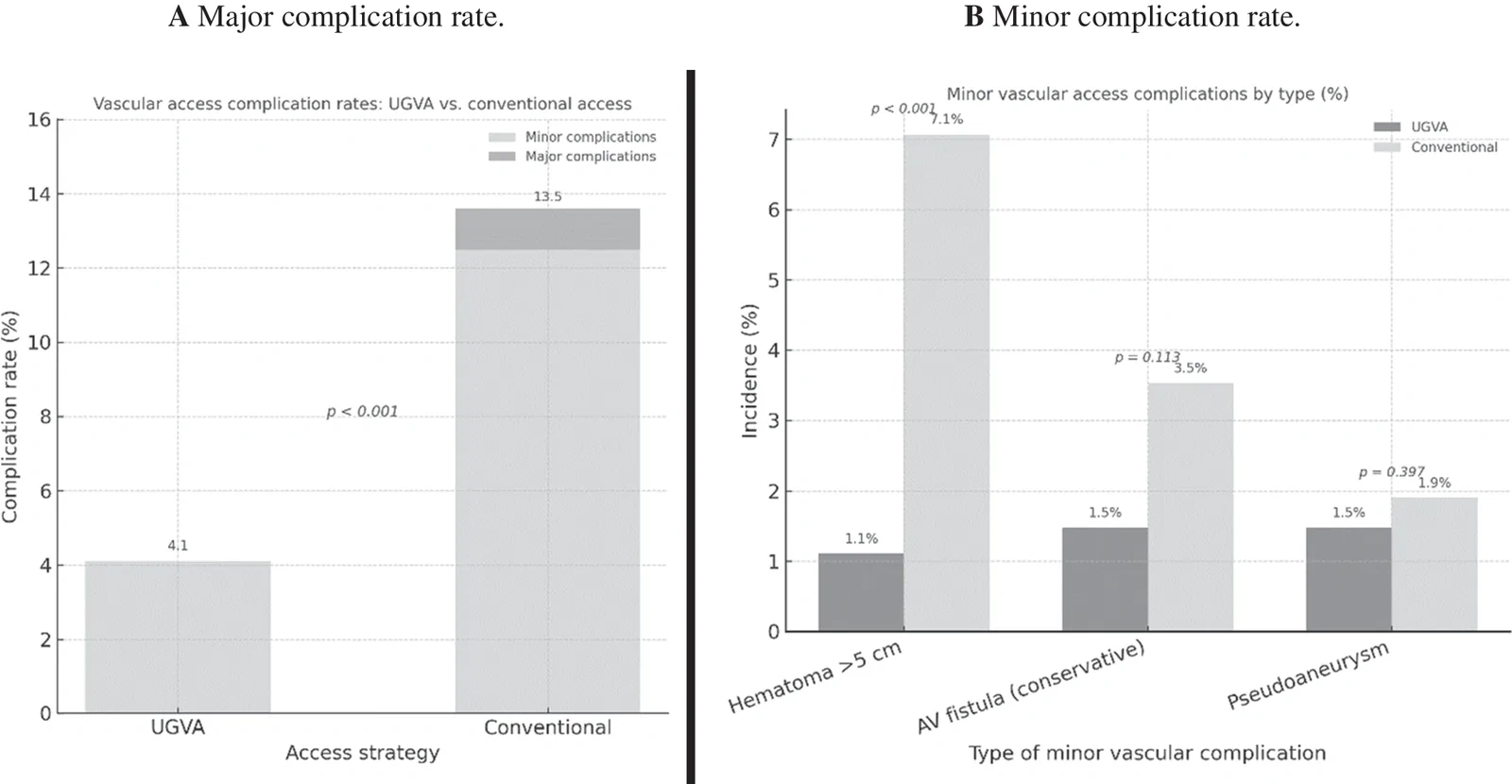
**A** Major complication rate. **B** Minor complication rate

Type and frequencies of vascular access complications are summarized in Table 3 and Fig. 1A, B. Major vascular access complications occurred in no case from the UGVA group and in 4 cases from the conventional group and included two cases of AV-fistulas requiring surgical revision and two cases of retroperitoneal hematoma requiring transfusion (UGVA 0/271 cases; 0% vs. conventional group 4/368 cases; 1.1%; *p* = 0.086). Minor vascular access complications were detected in 57/639 cases (8.9%). They occurred in 4.1% of the UGVA (11/271) vs. 12.5% of the conventional group (46/368) group (*p* < 0.001). These included hematomas > 5 cm (UGVA *n* = 3 vs. conventional group *n* = 26; *p* < 0.001), AV fistulas treated conservatively (UGVA group *n* = 4 vs. conventional *n* = 13; p = 0.113) and pseudoaneurysms treated by manual compression or ultrasound-guided thrombin injection (UGVA *n* = 4 vs. conventional *n* = 7; *p* = 0.397).

**Table 3:** Vascular access complications

	Total *n* = 639	UGVA *n* = 271	Conventional *n* = 368	*p*-value
**Total**	61 (9.5%)	11 (4.1%)	50 (13.5%)	< 0.001
**Major complications**				
AV fistula with surgical intervention	2 (0.3%)	0	2 (0.54%)	0.086
Retroperitoneal hematoma	2 (0.3%)	0	2 (0.3%)	0.086
**Minor complications**				
Hematoma > 5 cm	29 (4.5%)	3 (1.1%)	26 (7.1%)	< 0.001
Pseudoaneurysm treated conservatively or with thrombin injection	11 (1.7%)	4 (1.5%)	7 (1.9%)	0.397
AV fistula treated conservatively	17 (2.7%)	4 (1.5%)	13 (3.5%)	0.113

Overall, vascular access complications were significantly reduced using UGVA compared to the standard approach (4.1% (11/271) vs. 13.5% (50/368); *p* < 0.001). Complications were analyzed separately for first and repeat ablations. While rates tended to be higher in repeat procedures of the conventional cohort (14.9% vs. 10.3%), no such effect was seen in the UGVA group (4.8% vs. 3.9%).

We further analyzed vascular complications according to the type of vascular access. Among all 639 ablation procedures, 321 were performed with venous-only femoral access, and 318 with combined venous and arterial access. In both subgroups, ultrasound guidance was associated with fewer access-site complications (Table 4). In venous-only procedures, complications occurred in 10.2% of the conventional group and 3.1% of the UGVA group (*p* = 0.001). In procedures requiring arterial access, complication rates were 15.8% with conventional puncture versus 4.9% with UGVA (*p* < 0.001). These results indicate that UGVA provides a consistent safety benefit, particularly in procedures involving arterial puncture, where most major complications occurred in the conventional group.

**Table 4:** Subgroup analysis based on type of vascular access

Group	Venous-only complications (%)	Venous + arterial access (%)
Conventional	10.2%	15.8%
UGVA	3.1%	4.9%
*p*-value	0.001	< 0.001

### Temporal trend analysis (2014–2024)

**Fig. 2:**
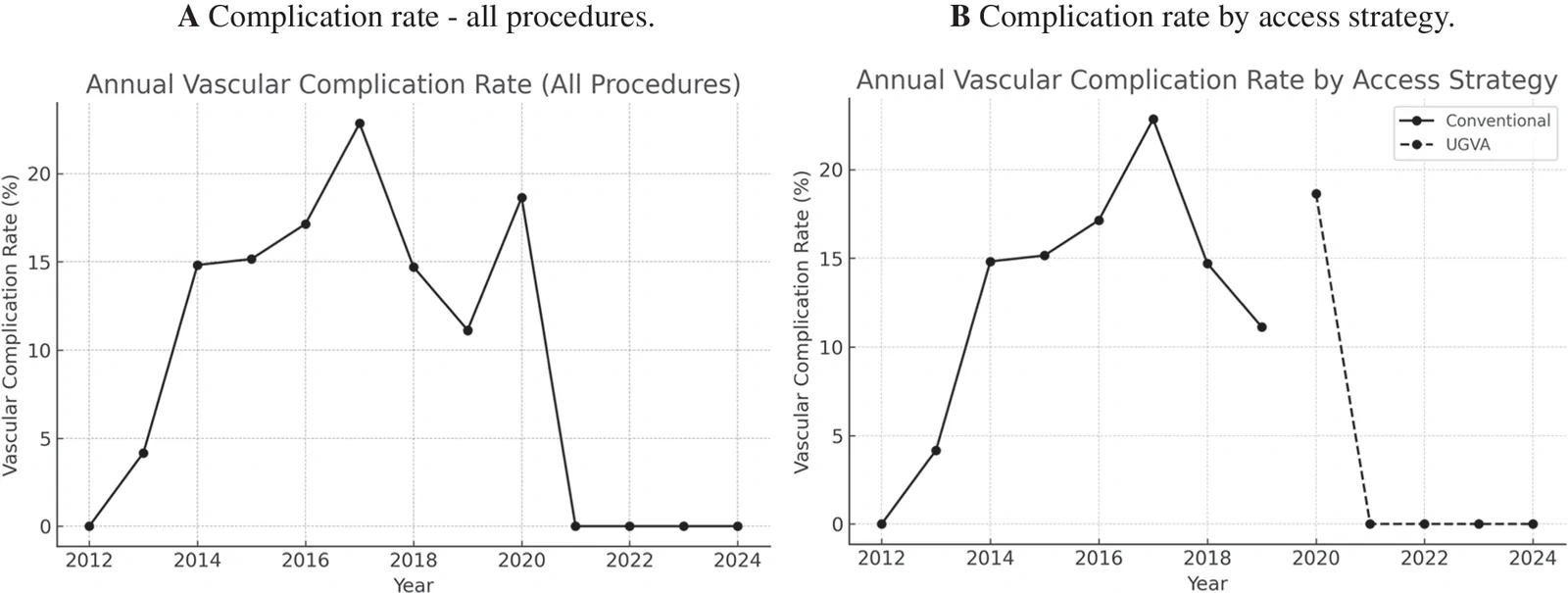
**A** Complication rate—all procedures. **B** Complication rate by access strategy

To address a potential learning-curve effect given the practice change in 2020, we analyzed annual vascular complication rates by access strategy from 2014 to 2024 (Fig. 2A, B). Rates in the conventional era (2014–2019) were relatively stable, while a stepwise decline was observed after the introduction of UGVA in 2020, with persistently lower annual rates for UGVA than for the contemporaneous conventional cohort where applicable. This supports an association with access strategy rather than incremental operator learning.

## Discussion

This large single-center analysis suggests that UGVA is associated with a substantially lower rate of vascular access complications in ACHD patients undergoing CA on uninterrupted OAC. The absolute risk reduction was ~ 9.4 percentage points (13.5% to 4.1%), driven primarily by fewer large hematomas and the absence of major complications in the UGVA cohort.

### Conventional vascular access

Vascular damage represents the major source of complications associated with CA of arrhythmias [17]. Conventional landmark-guided femoral puncture is inherently more prone to bleeding due to the lack of real-time vessel visualization. In patients without ACHD, these occur more frequently in patients on anticoagulant therapy, affected by obesity or diabetes mellitus and in female patients [16]. In patients with ACHD, femoral vascular access using a standard anatomical landmark-guided approach with modified Seldinger technique might be complicated by complex vascular anatomy or scarring of the femoral region due to previous catheter interventions or cardiac surgery. Conventional femoral venous access is performed by palpating the point of maximum pulsation of the femoral artery 1 cm below the inguinal ligament and pricking the vein 1 cm medially with respect to the artery. Studies in adult patients without ACHD showed that this approach is ineffective in up to 35% of cases with a percentage of complications up to 19% (including major femoral or retroperitoneal hematoma in 1.4%) [18]. In particular, a “low” venous puncture might generate pseudo-aneurysms or arteriovenous fistulas as some medial branches of the femoral artery run superficially to the femoral vein. On the other hand, a “high” venous puncture might carry the risk of retroperitoneal hematoma [17].

### Ultrasound-guided vascular access and comparison to the conventional approach

The effectiveness of different strategies in reducing femoral complications, in particular the use of smaller needles, but mainly the use of UGVA has been shown in previous studies [12–14].

The main strength of the ultrasound-guided technique is the ability to visualize size and depth of vascular structures thereby defining an optimal route of access. The technique is easy to use with a fast (< 10 cases) learning curve [18]. In a large single-center study, Sharma et al. [14] showed that UGVA for patients undergoing EP procedures for various cardiac arrhythmias was associated with a significantly lower 30-day risk of vascular access complications (5.3% vs. 1.1%; *p*= 0.002). Increased age and non-ultrasound vascular access were risk factors for vascular access complications. In the recently published prospective ULTRA-FAST trial with 320 consecutive AF ablation procedures, patients undergoing UGVA showed no significant difference in complication rates, but a shorter puncture time, fewer inadvertent arterial punctures and unsuccessful cannulations as well as fewer extra puncture attempts and higher first-pass success [19]. This effect was particularly evident in the subgroup of trainees. Yamagata [19] also stated that anatomical difficulties with position of the femoral vein directly above the artery (65% of patients overlap with the artery in the anteroposterior plane) can lead to inadvertent artery puncture and AV fistula. A retrospective analysis [12] reported a significant reduction in total vascular complications from 1.7% to 0.5% and a seven-fold reduction in the incidence of major vascular complications in the group of patients who underwent UGVA. Vascular complications in patients on OAC were reduced from 4.3% to 0.5%. In the study of Pellegrino [17], the percentage of major vascular complications was 0.3% (1 arteriovenous fistula) and the percentage of minor vascular complications was 0.3% (1 venous thrombosis). A systematic review and meta-analysis [21] with 7858 participants showed that the US-guided group had a significantly decreased incidence of total vascular complications compared to the non ultrasound-guided group (1.2 versus 3.2%) and the rate of major vascular complications was also reduced (0.7% versus 2%).

Compared to these studies, our overall complication rate was higher. This might be due to the fact that many ACHD patients present with challenging conditions like scarring around the puncture region and a routinely performed in-hospital ultrasound of the groin vessel on the first post-ablation day documented even minor complication. All above-mentioned studies performed post-ablation ultrasound only in highly symptomatic patients or documented only major complications (AV fistulas or pseudoaneurysms requiring intervention) [19].

Large randomized trials in non-ACHD patients, including FAUST [22], ULTRA-FAST [19], and UNIVERSAL [23], have shown an approximately twofold higher first-pass success and fewer total punctures with ultrasound compared to landmark techniques. Direct imaging also decreased the incidence of mispunctures and inadvertent arterial entries. In our cohort, no accidental arterial punctures occurred in the UGVA group, whereas ≈3% were observed in the conventional cohort, consistent with prior multicenter data demonstrating a fourfold reduction in wrong-vessel punctures with ultrasound guidance.

The year-by-year analysis of our cohort indicates that the reduction in vascular complications cannot be explained solely by a gradual learning effect or general procedural improvements over time. While later years showed a modest overall decline in complications, the distinct stepwise decrease coinciding with UGVA introduction and its independent association in multivariable analysis support a genuine technique-related benefit. Our patients in the UGVA group (treated from 2020 onward) underwent fewer ablation procedures per patient on average (~ 1.12) than those in the conventional group (2014–2019, ~ 1.57), which might be mainly due to the longer observation period of the earlier cohort. We cannot exclude that repeat procedures may lead to femoral vessel scarring and more challenging vascular access. However, ultrasound guidance resulted in a safer and more consistent vascular access in ACHD patients undergoing catheter ablation as initial or repeat procedure.

## Limitations

The main limitation is the retrospective nature of the study. To minimise selection bias, patients were enrolled consecutively and only patients on uninterrupted OAC were included. The baseline imbalances (OAC type, hypertension) may confound associations; we addressed these with sensitivity analyses. A significant difference in the use of VKA and DOAC was present between groups. To implement our findings in current practice and guidelines, further randomized controlled trials are requested. The propensity score matched dataset demonstrates that all measured baseline characteristics except arterial hypertension were well balanced. Because arterial hypertension was more common in the UGVA group, yet vascular access complications remained higher in the conventional group, it is unlikely that this residual imbalance explains the outcome differences. Thus, the observed reduction in vascular complications is more likely attributable to the use of ultrasound guidance rather than to differences in baseline patient characteristics.

The manuscript describes benefits of ultrasound guidance based on prior published evidence; however, procedural parameters such as puncture attempts, first-pass success, and time to access were not systematically recorded in this study and therefore cannot be confirmed by the present dataset.

## Conclusions

The use of UGVA in ACHD patients undergoing CA of atrial arrhythmias on uninterrupted oral anticoagulation significantly decreases vascular access complications and should therefore be recommended for routine use in clinical practice in this patient cohort.

## Supplementary Information

Below is the link to the electronic supplementary material.

ESM 1 (DOCX 15.3 KB)

## Abbreviations

UGVA: Ultrasound-guided vascular access
ACHD: Adult congenital heart disease
CA: Catheter ablation
CHD: Congenital heart disease
AF: Atrial fibrillation
OAC: Oral anticoagulation
DOAC: Direct oral anticoagulation
TSP: Transseptal puncture
ACT: Activated clotting time
VKA: Vitamin K antagonist
n.s.: Non-significant
