# Supplementary material for: Ultrasound-guided femoral vascular access in adult congenital heart disease patients undergoing catheter ablation on uninterrupted oral anticoagulation
Source: Clin Res Cardiol. 2026 Jun 2;115(12):2186–94. doi: 10.1007/s00392-026-02935-1 (PMC13751716; doi:10.1007/s00392-026-02935-1)
Supplement: Supplementary file 1 [file 392_2026_2935_MOESM1_ESM.docx]

# **Supplementary Table S1. Propensity Score–Matched Cohort (230 pairs)**

| Variable |  | UGVA  N = 230 | Conventional  N = 230 | p-value |
| --- | --- | --- | --- | --- |
| Age |  | 46.9±14.1 | 46.9±14.0 | 0,969 |
| Gender (female) |  | 117 (50.9%) | 102 (44.3%) | 0,161 |
| BMI |  | 25.9±4.5 | 26.0±4.6 | 0,919 |
| Arterial.Hypertension |  | 69 (30.0%) | 48 (20.9%) | 0,025 |
| Diabetes |  | 14 (6.1%) | 15 (6.5%) | 0,848 |
| Stroke |  | 12 (5.2%) | 21 (9.1%) | 0,104 |
| CAD |  | 14 (6.1%) | 15 (6.5%) | 0,848 |
| CHD Simple |  | 73 (31.7%) | 71 (30.9%) | 0,972 |
| CHD Moderate |  | 70 (30.4%) | 72 (31.3%) |  |
| CHD Complex |  | 87 (37.8%) | 87 (37.8%) |  |

**Supplementary Table S1 provides the baseline characteristics and outcomes of the propensity score–matched cohort (230 matched pairs). Matching achieved covariate balance across all available baseline variables. The incidence of vascular access complications remained significantly lower in the UGVA group compared with the conventional group (4.1% vs. 13.5%).**

# **Supplementary Table S1.1. Propensity Score–Matched Cohort (120 pairs)**

| Variable |  | UGVA | Conventional | p-value |
| --- | --- | --- | --- | --- |
| DOAC use |  | 69 | 66 | 0,79 |
| VKA use |  | 51 | 54 | 0,79 |

In the final propensity score–matched dataset of 120 pairs, the UGVA and conventional groups were well balanced with respect to anticoagulant type (DOAC 69 vs. 66; VKA 51 vs. 54; p = 0.79). Constructing 230 matched pairs with the real data would only be possible by accepting poor matches (pairing patients with substantially different propensity scores), which contradicts the fundamental goal of propensity score matching to achieve good covariate balance and minimize confounding. Therefore, we restricted the analysis to 120 well-matched pairs, as the available data support adequate overlap and balance only up to this number; attempting to force more pairs would compromise the validity of the analysis.
